# Engineering elastic bioactive composite hydrogels for promoting osteogenic differentiation of embryonic mesenchymal stem cells

**DOI:** 10.3389/fbioe.2022.1022153

**Published:** 2022-10-12

**Authors:** Min Wang, Yi Guo, Zexing Deng, Peng Xu

**Affiliations:** ^1^ Honghui Hospital, Xi’an Jiaotong University, Xi’an, China; ^2^ Shaanxi Key Laboratory of Brain Disorders, Shaanxi Key Laboratory of Ischemic Cardiovascular Disease, Institute of Basic and Translational Medicine, Xi’an Medical University, Xi’an, China; ^3^ College of Materials Science and Engineering, Xi’an University of Science and Technology, Xi’an, China

**Keywords:** composite hydrogels, polyitaconate, bioactive glass nanoparticles, mesenchymal stem cells, osteogenic differentiation

## Abstract

The development of bioactive materials with good mechanical properties and promotion of stem cell osteogenic differentiation has important application prospects in bone tissue engineering. In this paper, we designed a novel organic‒inorganic composite hydrogel (FPIGP@BGN-Sr) utilizing diacrylated F127 (DA-PF127), β-glycerophosphate-modified polyitaconate (PIGP) and strontium-doped bioactive glass nanoparticles (BGN-Sr) through free radical polymerization and coordination interactions and then evaluated its promoting effect on the osteogenic differentiation of mouse embryonic mesenchymal stem cells in detail. The results showed that the FPIGP@BGN-Sr hydrogel exhibited a controlled storage modulus by changing the amount of BGN-Sr. Notably, the FPIGP@BGN-Sr hydrogel possessed excellent elastic ability with a compressive strain of up to 98.6% and negligible change in mechanical properties after 10 cycles of compression. In addition, the FPIGP@BGN-Sr hydrogel had good cytocompatibility, maintained the activity and proliferation of mouse embryonic mesenchymal stem cells (C3H10T1/2), and effectively enhanced the activity of alkaline phosphatase, osteogenic gene expression and biomineralization ability of the cells. In conclusion, the excellent mechanical properties and osteogenic biological activity of the FPIGP@BGN-Sr hydrogel make it a promising organic‒inorganic composite bioactive material for stem cell-based bone regeneration.

## Introduction

Bone tissue defects, a common clinical disease with damaged bone structural integrity, are mainly caused by trauma, disease and surgery (Koons et al., 2020). The current methods of treating bone defects include autologous bone and allogeneic bone transplantation, tissue engineering technology and mesenchymal stem cell (MSC) therapy (Shang et al., 2021; Xue et al., 2022). As pluripotent cells, MSCs can self-renew and differentiate into osteoblasts, chondrocytes, adipocytes, and smooth muscle cells under specific inducing conditions, allowing for rapid and better tissue regeneration (Xia et al., 2018). In recent years, MSCs have been commonly used in bone repair and can be divided into bone marrow-derived mesenchymal stem cells (BMSCs), adipose-derived mesenchymal stem cells (AMSCs), embryonic-derived mesenchymal stem cells (EMSCs), buccal fat pad mesenchymal stem cells (BFP-MSCs) and dental pulp stem cells (DPSCs) (Moradi et al., 2018). Among these MSCs, EMSCs have the advantages of strong self-renewal ability, strong proliferation ability, multidirectional differentiation capacity, strong immunosuppressive effect, and convenient acquisition with large amounts (Saleh et al., 2020). Notably, C3H10T1/2 cells, as mouse EMSCs, can differentiate into osteoblasts under the stimulation of various growth factors and active substances, which exhibit potential therapeutic value in bone tissue regeneration (Lin et al., 2009; Liu et al., 2018; Jiang et al., 2019). Therefore, it is a key factor in effectively promoting the osteogenic differentiation of C3H10T1/2 cells in bone tissue engineering.

In recent years, bioactive glass (BG) has become a promising bioactive material in bone tissue engineering due to its good biocompatibility, biodegradability, and excellent osteoconductivity and osteoinductivity (Ribas et al., 2019). Previous studies have demonstrated that BGs can rapidly form hydroxyl carbonate-apatite on the surface after implantation *in vivo* to promote their conjugation with bone tissue, and the dissolved ions can upregulate the expression of osteoblast genes (Zeimaran et al., 2016). Notably, compared with macroscale BG-based biomaterials, nanoscale bioactive glass nanoparticles (BGNs) can significantly enhance the osteogenic differentiation of MSCs and bone tissue regeneration *in vivo* because of their biomimetic nano/microscale topology and high specific surface area (Guo et al., 2018; Zhu et al., 2020). In our previous studies, a series of BGNs were developed for the osteogenic differentiation of MSCs. For example, BGNs can enhance the osteogenic differentiation of ADSCs by activating the TGF-β/Smad3 signaling pathway (Guo et al., 2018). BGNs can effectively promote the osteogenic differentiation of BMSCs and accelerate bone tissue regeneration by loading miRNA (Xue et al., 2017). Furthermore, europium- or molybdenum-doped BGNs could significantly enhance the osteogenic differentiation of BMSCs and AMSCs, respectively (Li et al., 2018; Niu et al., 2019). Compared with europium and molybdenum, strontium (Sr) can induce bone matrix formation and inhibit osteoclastogenesis to reduce bone resorption, which exhibits significant potential application in bone tissue engineering (Kolodziejska et al., 2021). Encouragingly, the released Sr, calcium (Ca), and silicate in Sr-doped BGN have been proven to promote bone remodeling and regeneration through osteopromotive (Sr, Ca) and proangiogenic (silicate), as well as to inhibit osteoclastogenesis (Sr, silicate) through either the directly on osteoclasts or indirectly through osteoblasts (Lee et al., 2021).

The application of MSCs and BGNs in bone tissue engineering requires a specific scaffold to ensure that they can effectively reach the damaged site and sustainably release for a long time. Moreover, the scaffold needs to maintain cell activity and promote cell differentiation (Kroschwald et al., 2021; Dai et al., 2022). Among numerous scaffolds, biomedical hydrogels have been widely used because of their high-water content, 3D porous structure, tunable mechanical and degradation properties, and high similarity to the extracellular matrix (Mantha et al., 2019; Yue et al., 2020; Xue et al., 2021). Pluronic F-127 (PF127), a polyether with good biocompatibility, exhibits thermosensitive gel-forming capacity when dissolved in water at a certain concentration (Wang C. G. et al., 2019). In our previous studies, many thermosensitive PF127-based hydrogels have been prepared for application in tissue engineering, but they lack good elastic properties (Wang M. et al., 2019; Wang et al., 2020; Wang et al., 2021; Li et al., 2022). Previous reports indicated that the functionalization of PF127 using acryloyl chloride could prepare hydrogels with good mechanical properties (Shamma et al., 2022). Itaconic acid is a metabolite of the body, and its derivatives can regulate early inflammation in bone defects to promote osseointegration (Qiao et al., 2021). Sodium β-glycerophosphate (β-GP) has been proven to be an osteogenic supplement that can effectively promote the osteogenic differentiation of MSCs (He et al., 2019; Saravanan et al., 2019). Based on the above research background, it is necessary to develop an organic‒inorganic composite hydrogel scaffold based on Sr-doped BGNs and polyitaconate to promote the osteogenic differentiation of C3H10T1/2 cells. However, to our knowledge, no similar composite hydrogels have been studied.

**FIGURE 1:**
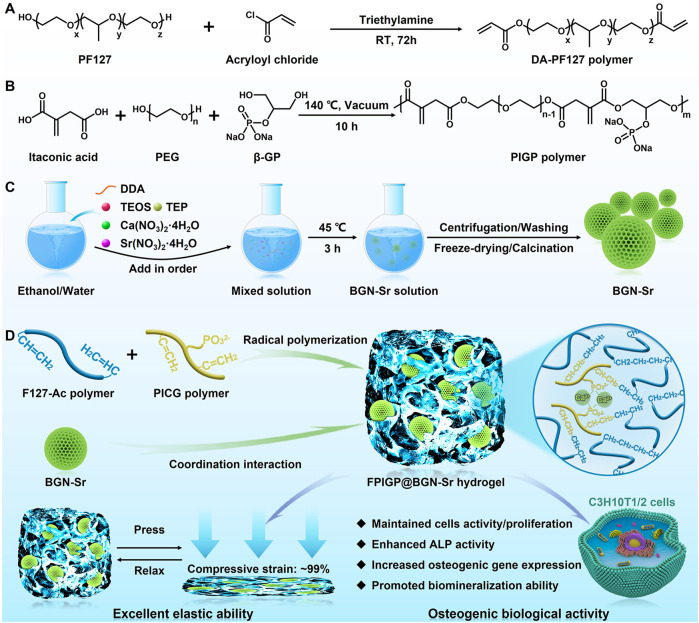
Schematic illustration showing the preparation of the FPIGP@BGN-Sr hydrogel to promote osteogenic differentiation of C3H10T1/2 cells. **(A)** The synthesis of DA-PF127 polymer. **(B)** The synthesis of PIGP polymer. **(C)** The fabrication of BGN-Sr. **(D)** The preparation of the FPIGP@BGN-Sr hydrogel and its application.

In this paper, we report an organic‒inorganic composite hydrogel (FPIGP@BGN-Sr) with favorable mechanical properties that promotes the osteogenic differentiation of C3H10T1/2 cells (Figure 1). The FPIGP@BGN-Sr hydrogel was formed from diacrylated PF127 (DA-PF127) polymer, β-GP-modified polyitaconate (PIGP) polymer and BGN-Sr through radical polymerization and coordination interactions. The DA-PF127 polymer was synthesized using PF127 and acrylic anhydride by a nucleophilic substitution reaction. The PIGP polymer was synthesized using itaconic acid, PEG and β-GP by esterification. The BGN-Sr was fabricated using various compounds containing silicon (Si), phosphorus (P), Ca or Sr by a sol-gel method. Subsequently, the physicochemical structure, mechanical properties, biocompatibility, and osteogenic differentiation of C3H10T1/2 cells including ALP activity, calcium deposition, and osteogenic gene expression were investigated in detail.

## Materials and methods

### Materials

Pluronic F-127 (PF127, E100P65E100, MW12600), itaconic acid, sodium β-glycerophosphate (β-GP) and triethylphosphate (TEP) were purchased from Sigma‒Aldrich. Acryloyl chloride, polyethylene glycol (PEG, MW1000), dodecylamine (DDA), tetraethyl orthosilicate (TEOS), calcium nitrate tetrahydrate (CN), strontium nitrate tetrahydrate (SrN) and 4-(2-hydroxyethoxy) phenyl-(2-hydroxy-2-propyl) ketone (Irgacure 2959) were obtained from Energy Chemical. Triethylamine and chloroform-d (CDCl_3_) were bought from Aladdin. Dulbecco’s modified Eagle’s medium (DMEM, GIBCO) and fetal bovine serum (FBS, GIBCO) were purchased from Life Technologies.

### Synthesis of polymers and hydrogels

#### Synthesis of DA-PF127 polymer

The diacrylated PF127 (DA-PF127) polymer was synthesized using PF127 and acryloyl chloride through the reaction of hydroxyl and acyl chloride according to the previous report (Choi et al., 2008). Briefly, 1 mmol PF127 and 5 mmol triethylamine were wholly dissolved in anhydrous dichloromethane and placed in an ice bath for 10 min. Subsequently, 5 mmol acryloyl chloride was slowly added dropwise to the above solution. After vigorous stirring at room temperature for 72 h under nitrogen, the mixed solution was evaporated to dryness and then dissolved in deionized water for purification by dialysis for 3 days. Finally, the DA-PF127 polymer was obtained by freeze-drying under a vacuum and stored in a 4°C refrigerator for later use.

#### Synthesis of PIGP polymer

The β-GP-modified polyitaconate (PIGP) polymer was synthesized using itaconic acid, PEG and β-GP by a melt-derived polymerization of carboxyl and hydroxyl groups. Briefly, 5 mmol PEG and 1 mmol β-GP were completely melted at 140°C under nitrogen and then 10 mmol itaconic acid was added and reacted at 140°C under vacuum for 10 h. The resulting PIGP polymer was obtained through dialysis purification for 3 days and freeze-dried under a vacuum, and stored in a 4°C refrigerator for later use.

#### Fabrication of BGN-Sr

Strontium (Sr)-doped bioactive glass nanoparticles (BGN-Sr) were fabricated using a DDA assisted sol-gel method according to our previous reports (Xue et al., 2019; Chen et al., 2022). Briefly, 1 g DDA was completely dissolved in a mixed solution comprised of 20 ml absolute ethanol and 6.25 ml deionized water at 45°C, and 0.5 ml TEOS was then slowly added dropwise to the above solution. After vigorous stirring for 30 min, 50.9 μl TEP, 264.7 mg CN and 63.6 mg SrN were added to the above solution in order every 30 min. The mixed solution was vigorously stirred at 45°C for 2.5 h. The resulting BGN-Sr was purified by centrifugation, washed with ethanol and water, vacuum freeze-dried and calcined at high temperatures including 350°C for 3 h and 650°C for 3 h. The resulting BGN-Sr was stored in a 4°C refrigerator for later use. The theoretical molar composition of BGN-Sr was 60SiO_2_: 30CaO: 4P_2_O_5_: 6SrO according to the above feeding ratio.

#### Preparation of FPIGP@BGN-Sr hydrogel

The FPIGP@BGN-Sr hydrogel was prepared using DA-PF127, PIGP and BGN-Sr through double cross-linking including the free radical copolymerization of double bonds and the coordination interaction between phosphate and calcium ions. Briefly, 10% (wt/vol) DA-PF127 and 2.5% (wt/vol) PIGP polymers were completely dissolved in deionized water at 4°C, and BGN-Sr with different concentrations (0, 25, 50 and 100 μg/ml) were then added to the above solution separately and vigorously stirred for 5 min. Finally, the solutions were polymerized through an ultraviolet lamp for 1 min after adding 0.1% (wt/vol) Irgacure 2959 to obtain the FPIGP@BGN-Sr hydrogels named FPIGP, FPIGP@BGN-Sr-1 (BGN-Sr: 25 μg/ml), FPIGP@BGN-Sr-2 (BGN-Sr: 50 μg/ml) and FPIGP@BGN-Sr-3 (BGN-Sr: 100 μg/ml), respectively.

### Physicochemical structure of polymers and hydrogels

The physicochemical structures of original raw materials, DA-PF127 and PIGP polymers were analyzed using a ^1^H nuclear magnetic resonance instrument (^1^H NMR, Ascend 400 MHz, Bruker) after dissolving in CDCl_3_. Moreover, the Fourier infrared spectroscopy of polymers, BGN-Sr and hydrogel were also measured through the Fourier transformation infrared spectrometer (FTIR, ALPHA II, Bruker) in the range of 4000 to 650 cm^−1^. The chemical structure of BGN-Sr was analyzed by X-ray diffractometer (XRD, D/MAX-RB, Rigaku) at 40 kV and 30 mA. In addition, the morphology and energy dispersive spectroscopy (EDS) of the BGN-Sr and FPIGP@BGN-Sr hydrogels were observed and measured through scanning electron microscopy (SEM, GeminiSEM 500, Zeiss) and transmission electron microscopy (TEM, JEM-2100Plus, JEOL).

### Physicochemical properties of hydrogels

The physicochemical properties including elasticity, storage modulus (G′), loss modulus (G″) and viscosity of FPIGP@BGN-Sr hydrogels were evaluated by a TA rheometer (DHR-2) under different conditions. Briefly, the stress of the FPIGP and FPIGP@BGN-Sr hydrogels were detected along with the increase from 0% to 99% compressive strain and the change between 0% and 60% compressive strain for 10 cycles at 25°C, respectively. The G′ and G″ of the FPIGP and FPIGP@BGN-Sr hydrogels were measured under 1 Hz frequency and 1% oscillation strain. In addition, the viscosity of FPIGP and FPIGP@BGN-Sr hydrogels were measured along with the increase from 1 1/s to 100 1/s shear rate at 25°C.

The degradation and BGN-Sr nanoparticle release of the FPIGP@BGN-Sr hydrogel were evaluated by detecting the content change of the hydrogel and BGN-Sr. Briefly, 0.2 ml FPIGP@BGN-Sr hydrogel was placed in a container with one end open and then in 2 ml phosphate buffer solution (PBS, pH 7.4). The residual weight of the hydrogel was detected to calculate the degradation rate during 20 days. In addition, the content of BGN-Sr was also measured by inductively coupled plasma mass spectrometer (ICP-MS, NexION^™^ 350D, PerkinElmer, United States) to calculate the release rate of BGN-Sr nanoparticles during degradation.

### Hemocompatibility of hydrogels

The hemocompatibility of the FPIGP and FPIGP@BGN-Sr hydrogels was investigated by detecting the hemolysis ratios and observing the erythrocyte integrity. Briefly, 0.1 ml FPIGP and FPIGP@BGN-Sr hydrogels were placed at the bottom of the centrifuge tube and 0.5 ml 5% erythrocyte suspension was added and incubated for 30 min at 37°C. Subsequently, the hemolysis of erythrocytes was observed and the absorbance of the supernatant was detected using a microplate reader (SpectraMax i3, Molecular Devices) at 540 nm after centrifuging at 1000 rpm for 5 min. Moreover, the erythrocyte integrity was observed by a fluorescence microscope (BX51, Olympus). PBS and 1% Triton X-100 were used as the control groups.

### Cell culture

Mouse embryonic mesenchymal stem cells (C3H10T1/2) and murine-derived macrophage cell lines (RAW 264.7) were purchased from the Cell Bank of the Chinese Academy of Sciences (Shanghai, China). The cells were cultured in basal medium composed of DMEM, 15% (vol/vol) FBS, 100 U/ml penicillin and 100 μg/ml streptomycin, and placed in a cell incubator with a 5% carbon dioxide humidified atmosphere at 37°C. The basal medium was changed every 2 days, and the cells were passaged when they had grown to 80% of the bottom area. The osteoinductive medium was composed of basal medium, 100 nM dexamethasone, 10 mM β-GP and 50 μg/ml ascorbic acid.

### Cytotoxicity and anti-inflammatory ability of hydrogels

The cytotoxicity of hydrogels was investigated by a cell counting kit-8 (CCK8) assay and live-dead staining of C3H10T1/2 cells. Briefly, the cells were seeded in 96-well cell culture plates (CostarTM, Corning) at a density of 1500 cells/well and cultured in basal medium (150 μl) for 8 h. All hydrogels were soaked in basal medium for 12 h to remove residual monomers and Irgacure 2959. Subsequently, the original basal medium was discarded, an equal volume of FPIGP, FPIGP@BGN-Sr-1, FPIGP@BGN-Sr-2 and FPIGP@BGN-Sr-3 hydrogels (50 μl) were added to direct contact with the cells, and then 150 μl basal medium was added to each well. After 1-, 3- and 5-day cocultivation, the original basal medium was replaced by basal medium containing 10% CCK8 and further incubated for 4 h. Finally, the absorbance was measured at 450 nm using a microplate reader to evaluate the cell viability. Moreover, after 1 and 5 days of cocultivation, the cells were stained with a live-dead cell staining kit and further observed using a fluorescence microscope (BX51, Olympus). The control group stands for no hydrogel added during cell culture.

In addition, the anti-inflammatory ability of the FPIGP@BGN-Sr hydrogel was evaluated by detecting the expression of inflammation-related genes. Briefly, RAW264.7 cells were seeded in 12-well cell culture plates at a density of 40,000 cells/well and cultured in basal medium without penicillin and streptomycin for 24 h. The cells were treated with lipopolysaccharide (LPS, 100 ng/ml) for 24 h, and then the dexamethasone (DEX), F@BGN-Sr hydrogel (DA-PF127 and BGN-Sr) and FPIGP@BGN-Sr hydrogel were added. After 2 days of cocultivation, the mRNA expression (*IL-6*, *IL-1β* and *Tnf-α*) was detected by the quantitative real-time polymerase chain reaction (qRT‒PCR).

### Osteogenic differentiation of C3H10T1/2

#### ALP activity evaluation

Alkaline phosphatase (ALP) activity can be used to evaluate the early osteogenic differentiation of C3H10T1/2 cells. Briefly, C3H10T1/2 cells were seeded in 24-well cell culture plates (CostarTM, Corning) at a density of 30,000 cells/well and cultured with 1.5 ml basal medium until approximately 80% confluence. All hydrogels were soaked in basal medium for 12 h to remove residual monomers and Irgacure 2959. Similarly, the original basal medium was removed and an equal volume of FPIGP, FPIGP@BGN-Sr-1, FPIGP@BGN-Sr-2 and FPIGP@BGN-Sr-3 hydrogels (150 μl) were added to direct contact with the cells, and then 1.5 ml osteoinductive medium was added to each well. The medium was changed every 3 days during the culture. As an early marker of osteogenic differentiation, ALP activity was quantitatively and qualitatively detected on days 7 and 14, respectively. The fluorescence and the total protein content of cell lysates were measured by the Anaspec sensolyte pNPP ALP assay kit (Fremont, CA) at 450 nm and BCA protein assay kit (Thermo Fisher Scientific) respectively, which further calculated ALP activity. In addition, the qualitative analysis was carried out with an alkaline phosphatase staining (azo coupling method) kit (Solarbio). After the induced cells were fixed with ALP fixative and stained with ALP staining solution, the relative grayscale analysis of the ALP staining results was performed using ImageJ software. The control group stands for no hydrogel added during cell culture.

#### Calcium deposition staining

The calcium deposition and extracellular matrix mineralization were assessed with an alizarin red staining (ARS) kit (GenMed). Briefly, C3H10T1/2 cells were cultured in osteoinductive medium containing four kinds of hydrogels. On the 21st day, the cells were fixed with 4% paraformaldehyde fixative and then stained with alizarin red staining solution at room temperature for 10 min. The relative grayscale analysis of the alizarin staining results was performed using ImageJ software. The control group stands for no hydrogel added during cell culture.

#### Osteogenic gene expression

The osteogenic gene (*Runx2* and *Bsp*) expression was measured by qRT‒PCR. Briefly, the C3H10T1/2 cells were cultured in the osteoinductive medium containing four kinds of hydrogels. After 7 and 14 days of cocultivation, the cells were lysed using Trizol reagent (Life Technologies) to extract the total RNA, which was further reverse transcribed into cDNA using an all-in-one RT MasterMix (ABM, G490) reverse transcription kit. Subsequently, *Runx2* and *Bsp* expression was measured by iTaq Univer SYBR Green Supermix and a fast real-time PCR system (Applied Biosystems 7500), and the relative gene expression was further calculated using the 2^−ΔΔCt^ method. Glyceraldehyde-3-phosphate dehydrogenase (GAPDH) was used as a housekeeping gene. The control group stands for no hydrogel added during cell culture.

### Statistical analysis

All data are expressed as the mean ± standard deviation. Significant differences between groups were determined by Student’s *t*-test. *p < 0.05 and **p < 0.01 indicate significant differences.

## Results

### Synthesis and characterization of polymers and hydrogel

The FPIGP@BGN-Sr hydrogels were prepared using DA-PF127, PIGP and BGN-Sr at different concentrations through free radical copolymerization between DA-PF127 and PIGP polymer and coordination interactions between PIGP and BGN-Sr. For each component of the FPIGP@BGN-Sr hydrogel, the DA-PF127 polymer was synthesized using PF127 and acryloyl chloride through a nucleophilic substitution reaction. The PIGP polymer was synthesized using itaconic acid, PEG and β-GP through an esterification reaction. The BGN-Sr was fabricated using TEOS, TEP, CN and SrN through the DDA assisted sol-gel method (Figures 1A–C).

**FIGURE 2:**
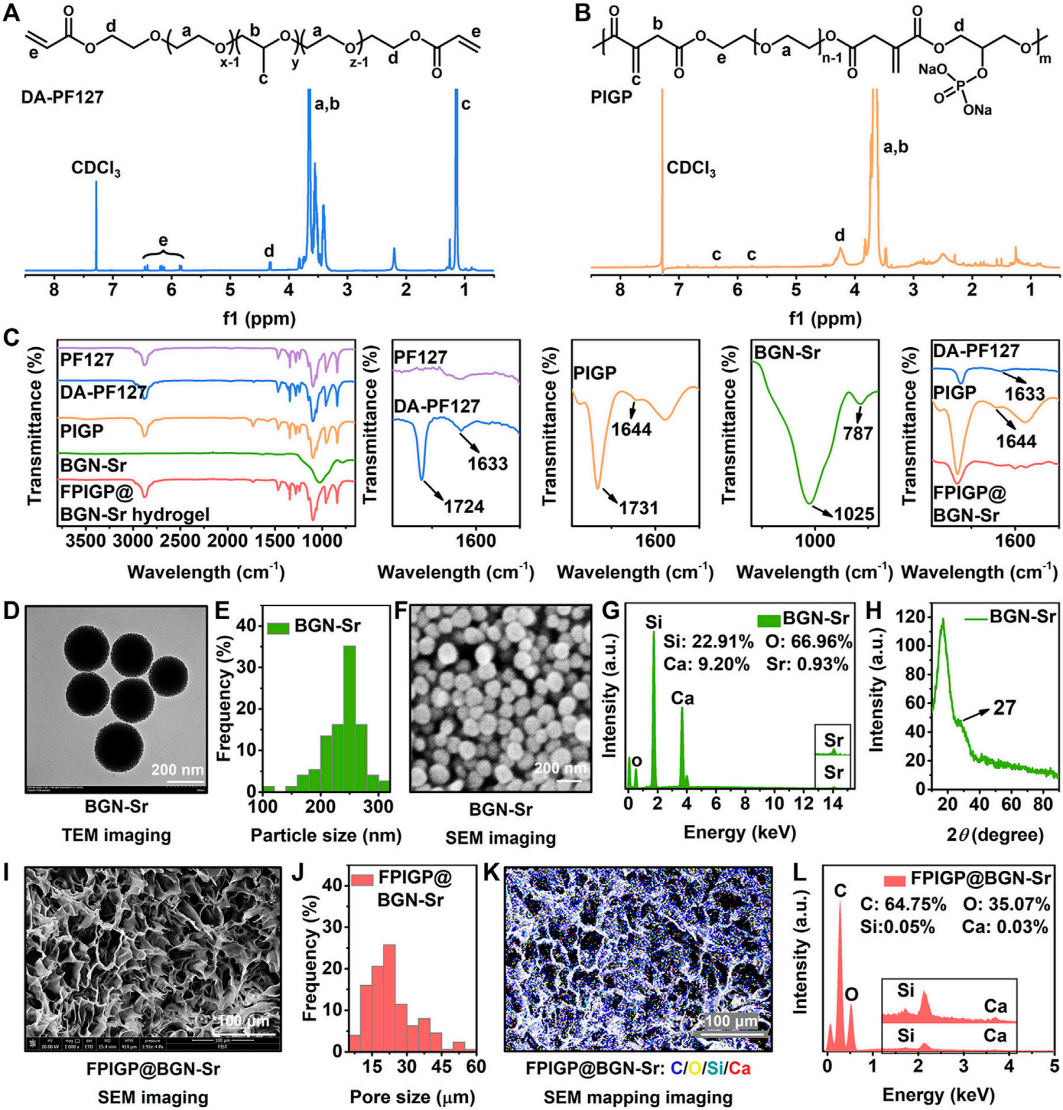
Physicochemical structures of polymers and hydrogels. **(A**,**B)** The ^1^H NMR spectra of **(A)** DA-PF127 polymer and **(B)** PIGP polymer. **(C)** The FTIR spectra of PF127, DA-PF127 and PIGP polymer, BGN-Sr and FPIGP@BGN-Sr hydrogel. **(D)** The TEM image of BGN-Sr. **(E)** The particle size of BGN-Sr. **(F)** The SEM image of BGN-Sr. **(G)** The EDS spectrum of BGN-Sr. **(H)** The XRD pattern of BGN-Sr. **(I)** The SEM image of FPIGP@BGN-Sr hydrogel. **(J)** The pore size of FPIGP@BGN-Sr hydrogel. **(K)** The EDS mapping of FPIGP@BGN-Sr hydrogel. **(L)** The EDS spectrum of FPIGP@BGN-Sr hydrogel.

The physicochemical structures of the original raw materials, polymers, nanoparticles and hydrogels were identified through ^1^H NMR, FTIR, XRD, TEM and SEM (Figure 2 and Supplementary Figure S1). According to the ^1^H NMR spectrum of the original raw materials, DA-PF127 and PIGP polymers, the peaks at 7.28 ppm and 4.70 ppm were assigned to the solvent peaks of CDCl_3_ and D_2_O, respectively. The multiple peaks at approximately 3.60 ppm and the peak at 1.13 ppm were attributed to the methylene (-CH_2_-) and methyl (-CH_3_) groups of the PF127 polymer, respectively. The three peaks between 5.80 ppm and 6.50 ppm belonged to the ethylene union (-CH=CH_2_) of acryloyl chloride. The peak of 4.33 ppm was identified as the methylene (-COO-CH_2_-) attached to the newly formed ester bond of the DA-PF127 polymer. Therefore, the appearance of the above characteristic peaks indicated the successful synthesis of the DA-PF127 polymer (Figure 2A and Supplementary Figure S1A). The multiple peaks at approximately 3.65 ppm were regarded as the methylene (-CH_2_-) of PEG and itaconic acid. The double peaks of 5.75 ppm and 6.36 ppm belonged to the ethylene union (-C=CH_2_) of itaconic acid. The peak of 4.26 ppm was identified as the methylene (-COO-CH_2_-) attached to the newly formed ester bond of the PIGP polymer. Therefore, the appearance of the above characteristic peaks suggested the successful synthesis of the PIGP polymer (Figure 2B and Supplementary Figures S1B–D). In the FTIR results, compared with PF127, the peaks at 1633 cm^−1^ and 1724 cm^−1^ were respectively assigned to the ethylene union (-CH=CH_2_) and the newly formed ester bond (-COO-) of the DA-PF127 polymer, which indicated the successful synthesis of the DA-PF127 polymer (Sosnik et al., 2003). The peaks at 1644 cm^−1^ and 1731 cm^−1^ belonged to the ethylene union (-C=CH_2_) and the newly formed ester bond (-COO-) of the PIGP polymer, respectively, suggesting the successful synthesis of the PIGP polymer. The peaks at 787 cm^−1^ and 1025 cm^−1^ were assigned to the Si-O-Si of BGN-Sr nanoparticles (Niu et al., 2021). Compared with DF-F127 and PIGP polymers, the disappearance of peaks at 1644 cm^−1^ and 1731 cm^−1^ indicated that the FPIGP@BGN-Sr hydrogel was successfully fabricated through free radical copolymerization (Figure 2C). Additionally, the BGN-Sr nanoparticles had a superficially porous spherical structure and exhibited good dispersibility in water without apparent aggregation (Figure 2D). The particle size of BGN-Sr was approximately 250 nm according to the statistical analysis of the TEM result (Figure 2E). It can also be clearly observed from the SEM image that the BGN-Sr nanoparticles had a spherical structure with a relatively uniform size (Figure 2F). The EDS result showed that the atomic percentages of BGN-Sr nanoparticles were Si (22.91%), O (66.96%), Ca (9.20%) and Sr (0.93%), suggesting the successful formation of BGN-Sr nanoparticles (Figure 2G). In the XRD patterns, there were no diffraction peaks except for amorphous silica at 27°, which indicated that the doping of Sr did not affect the amorphous structure of nanoparticles (Figure 2H). Moreover, the FPIGP@BGN-Sr hydrogel exhibited a typical porous structure (Figure 2I). The pore size of the FPIGP@BGN-Sr hydrogel was approximately 20 μm according to the statistical analysis of the SEM image (Figure 2J). According to the EDS mapping of the FPIGP@BGN-Sr hydrogel, it is obvious that BGN-Sr nanoparticles were evenly distributed within the whole hydrogel (Figure 2K). Unfortunately, only Si and Ca can be detected in the EDS results of the FPIGP@BGN-Sr hydrogels owing to the small doping content of BGN-Sr nanoparticles (Figure 2L). In conclusion, the above results demonstrated the successful preparation of DA-PF127 and PICG polymers, BGN-Sr nanoparticles and FPIGP@BGN-Sr hydrogels.

### Physicochemical properties of hydrogels

**FIGURE 3:**
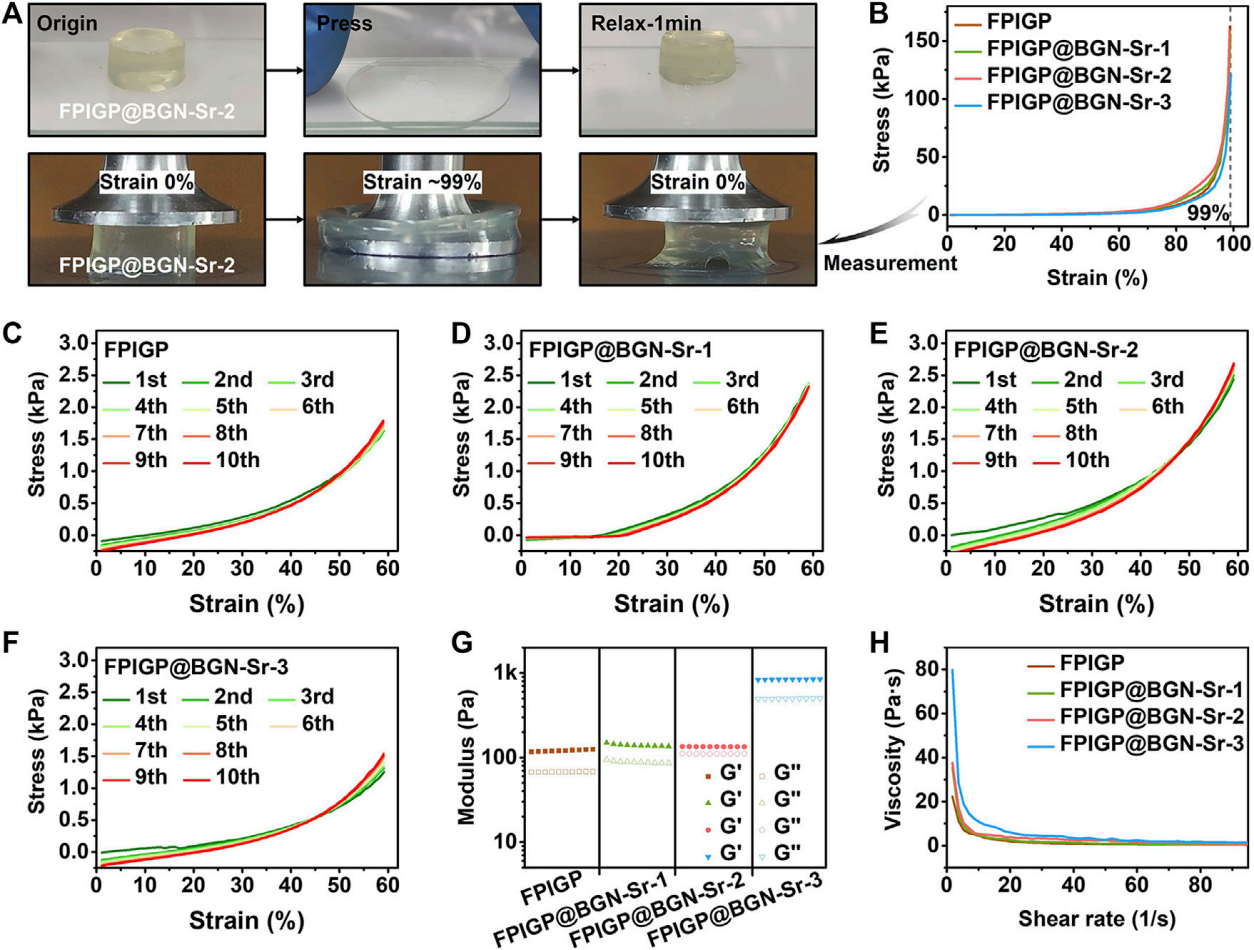
Physicochemical properties of hydrogels. **(A)** The photographs of FPIGP@BGN-Sr-2 hydrogel before and after being squeezed between two glass sheets. **(B)** The stress‒strain curves of FPIGP and FPIGP@BGN-Sr hydrogels. **(C**–**F)** The stress‒strain curves of **(C)** FPIGP hydrogel, **(D)** FPIGP@BGN-Sr-1 hydrogel, **(E)** FPIGP@BGN-Sr-2 hydrogel and **(F)** FPIGP@BGN-Sr-3 hydrogel within 10 cycles at 60% strain. **(G)** The G′ and G″ of FPIGP and FPIGP@BGN-Sr hydrogels. **(H)** The viscosity of FPIGP and FPIGP@BGN-Sr hydrogels with a shear rate from 1 to 100 1/s.

The stress, modulus and viscosity were measured to evaluate the physicochemical properties of the FPIGP@BGN-Sr hydrogels (Figure 3). The FPIGP@BGN-Sr-2 hydrogel was placed in the middle of two glass slides, and the compressive strain of the hydrogel was maximized by pressing the upper glass slides. After the upper glass was removed for 1 min, the hydrogel could almost return to its original state, indicating that the hydrogel had good elastic resilience (Figure 3A). Subsequently, the mechanical properties of the hydrogels were examined in detail using a TA rheometer. The compressive stress of all hydrogels increased with increasing compressive strain, and no decrease was observed when the compressive strain reached 99%, indicating that all hydrogels exhibited good elastic properties without being crushed. When the compressive strain was 99%, the compressive stress of each hydrogel was FPIGP: 162.56 kPa, FPIGP@BGN-Sr-1: 155.90 kPa, FPIGP@BGN-Sr-2: 159.10 kPa and FPIGP@BGN-Sr-3: 121.02 kPa, respectively. The above results indicated that all hydrogels possessed good compressive elasticity, and the addition of BGN-Sr nanoparticles did not affect the compressive strain of the hydrogels but had a weak effect on the maximum compressive stress (Figure 3B and Supplementary Video S1). The elastic resilience of each hydrogel was evaluated by cyclic compressive tests. The compressive curve of all hydrogels exhibited negligible change with hysteresis after 10 cycles when the compressive strain was 60%. The first cycle compressive stress of each hydrogel was FPIGP: 1.63 kPa, FPIGP@BGN-Sr-1: 2.36 kPa, FPIGP@BGN-Sr-2: 2.43 kPa and FPIGP@BGN -Sr-3: 1.25 kPa. The tenth cycle compressive stress of each hydrogel was FPIGP: 1.79 kPa, FPIGP@BGN-Sr-1: 2.32 kPa, FPIGP@BGN-Sr-2: 2.68 kPa and FPIGP@BGN-Sr-3: 1.51 kPa. The above results showed that all hydrogels had good resilience, and the BGN-Sr nanoparticles did not affect the resilience of the hydrogels but had a greater impact on their compressive stress when the compressive strain was 60% (Figures 3C–F). The G′ of each hydrogel was FPIGP: 120.90 Pa, FPIGP@BGN-Sr-1: 140.11 Pa, FPIGP@BGN-Sr-2: 134.62 Pa and FPIGP@BGN -Sr-3: 840.44 Pa, respectively. The results indicated that the low content (25 and 50 μg/ml) of BGN nanoparticles did not affect the G′ of hydrogels, while the high content (100 μg/ml) of BGN nanoparticles significantly increased the G′ of hydrogels (Figure 3G). In addition, the viscosity of all hydrogels decreased gradually with increasing shear rate from 1 1/s to 100 1/s, indicating that the hydrogels possessed a certain shear-thinning behavior to provide the possibility for further injectable demonstration (Figure 3H). With increasing degradation time, the weight of the FPIGP@BGN-Sr hydrogel gradually decreased. The degradation velocity of the FPIGP@BGN-Sr hydrogel was faster within 7 days, and then gradually became slower. After degradation for 12 days, the cumulative degradation rate of the FPIGP@BGN-Sr hydrogel exceeded 75% (Supplementary Figure S2A). In addition, the hydrogel exhibited sustained release of BGN-Sr nanoparticles, and the cumulative release rate of BGN-Sr was approximately 60% after degradation for 12 days (Supplementary Figure S2B). In conclusion, the above results demonstrated that the addition of BGN-Sr nanoparticles can affect the mechanical properties of the FPIGP@BGN-Sr hydrogel without destroying its good elastic resilience, and the FPIGP@BGN-Sr hydrogel possessed degradation and sustained release of BGN-Sr nanoparticles.

### Biocompatibility and anti-inflammatory ability evaluation of hydrogels

**FIGURE 4:**
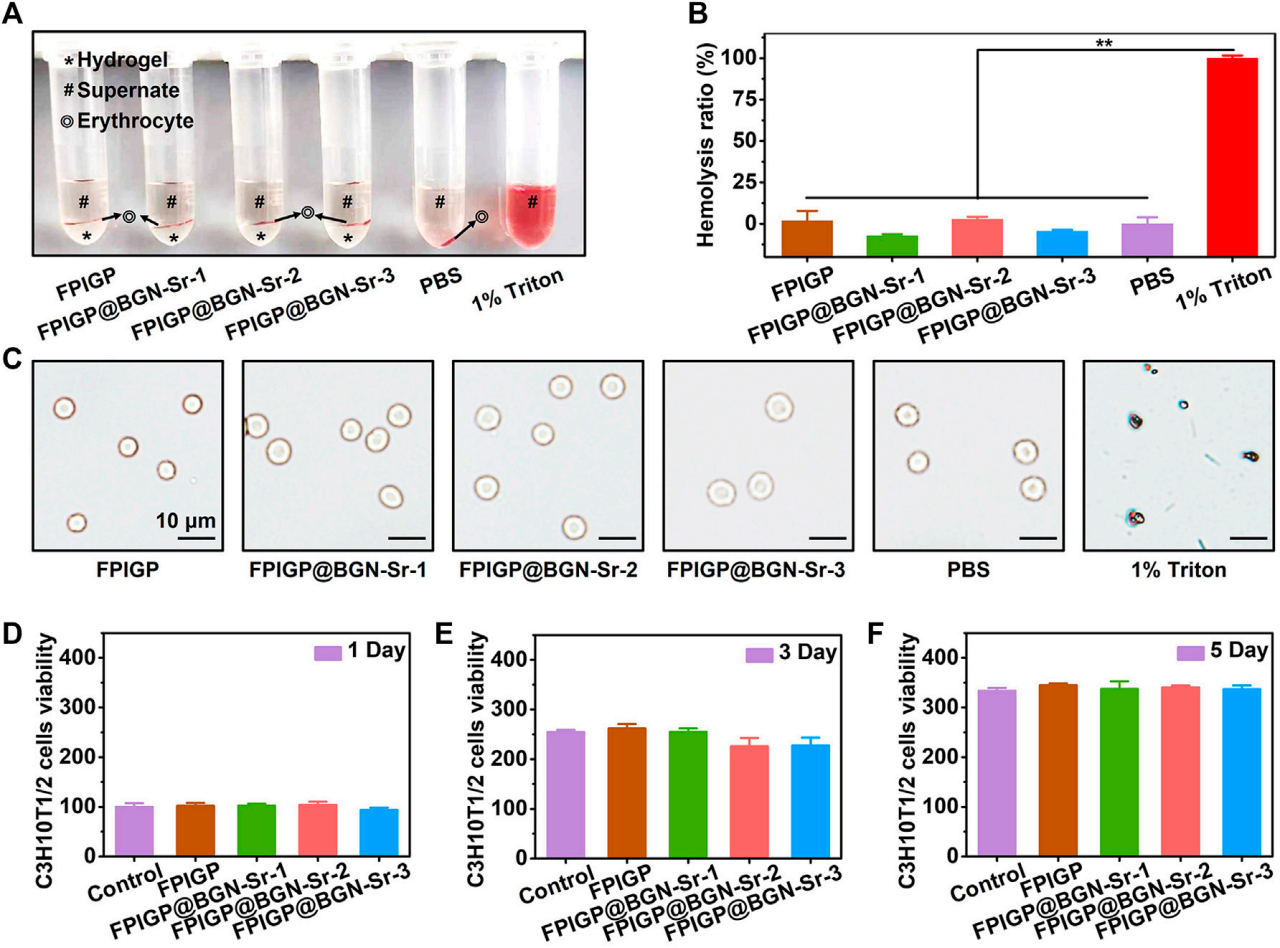
Biocompatibility evaluation of hydrogels. **(A)** The optical picture of erythrocytes after incubating with hydrogels. **(B)** The hemolysis ratio after incubating with hydrogels. **(C)** The morphology of erythrocyte after incubating with hydrogels. **(D**–**F)** The C3H10T1/2 cells viability after incubating with hydrogels for **(D)** 1 day, **(E)** 3 days and **(F)** 5 days.

The biocompatibility of the FPIGP and FPIGP@BGN-Sr hydrogels was evaluated by investigating their hemocompatibility and cytocompatibility (Figure 4). The hemocompatibility of the FPIGP and FPIGP@BGN-Sr hydrogels was investigated by detecting the hemolysis ratios and observing the erythrocyte integrity. For the erythrocyte suspension obtained by centrifugation after cocultivation with the PBS, hydrogels or Triton for 30 min at 37°C, no obvious erythrocyte precipitation was observed and the supernatant was uniform and bright red in the Triton group, indicating that the erythrocytes had been destroyed with severe hemolysis. In contrast, obvious erythrocyte precipitation was observed on the surface of all hydrogels and their supernatants were clear and transparent, which indicated that the erythrocytes were not severely damaged and that no hemolysis occurred (Figure 4A). The hemolysis ratios were calculated by detecting the absorbance of the supernatant. There were no significant differences in the hemolysis rate among the PBS and all hydrogels, and their hemolysis ratios were all less than 5% compared with Triton (100%), suggesting that all hydrogels caused negligible damage to erythrocytes (Figure 4B). Moreover, compared with the severely damaged erythrocytes in the Triton group, the erythrocytes with intact status in the all hydrogels and PBS groups were observed, which further indicated that the erythrocytes were not damaged (Figure 4C). The above results showed that the FPIGP and FPIGP@BGN-Sr hydrogels had good hemocompatibility.

The cytocompatibility of the FPIGP and FPIGP@BGN-Sr hydrogels was evaluated by measuring the viability of C3H10T1/2 cells cocultivated with the hydrogels after 1, 3, and 5 days. Based on the cell viability (100%) of the control group when cocultivated for 1 day. The cell viability of each hydrogel was FPIGP: 102.11%, FPIGP@BGN-Sr-1: 102.48%, FPIGP@BGN-Sr-2: 104.28% and FPIGP@BGN-Sr-3: 93.36%. Compared with the control group, the cell viability of each hydrogel was maintained at approximately 100% at 1 day, and there were no significant differences among them, indicating that the hydrogels can maintain cell viability and that the addition of BGN-Sr nanoparticles cannot cause cytotoxicity (Figure 4D). After coincubation for 3 and 5 days, the cell viability of the control group reached 254.65% and 333.69%, respectively, and the cell viability of each hydrogel was FPIGP: 261.85% and 344.91%, FPIGP@BGN-Sr-1: 255.03% and 337.47%, FPIGP@BGN-Sr-2: 226.11% and 340.26% and FPIGP@BGN-Sr-3: 227.54% and 336.72%, respectively. The cell viability of all groups increased with coincubation time, and no significant differences were observed, indicating that the hydrogels can maintain cell proliferation (Figures 4E,F). In addition, compared with the control group, the cells exhibited a good growth state and no significant death after incubation with the FPIGP and FPIGP@BGN-Sr hydrogels for 1 and 5 days, further indicating that the FPIGP and FPIGP@BGN-Sr hydrogels did not cause serious damage to the cells (Supplementary Figure S3). The above results indicated that the FPIGP and FPIGP@BGN-Sr hydrogels had good cytocompatibility.

Moreover, the anti-inflammatory ability of the FPIGP@BGN-Sr hydrogel was evaluated by detecting the expression of inflammation-related genes including *IL-6*, *IL-1β* and *Tnf-α*. After treatment with LPS for 24 h, RAW 264.7 cells exhibited increased *IL-6*, *IL-1β* and *Tnf-α* expression, suggesting the construction of inflammation. Compared with the F@BGN-Sr hydrogel, the DEX and FPIGP@BGN-Sr hydrogel groups significantly downregulated *IL-6* expression, indicating that the addition of PIGP polymer effectively downregulated *IL-6* expression (Supplementary Figure S4A). For *IL-1β* and *Tnf-α* expression, no significant difference was observed between the F@BGN-Sr hydrogel and FPIGP@BGN-Sr hydrogel groups, indicating that the addition of PIGP polymer had less impact on the *IL-1β* and *Tnf-α* expression (Supplementary Figures S4B,C). The above results suggest that the FPIGP@BGN-Sr hydrogel might possess anti-inflammatory ability by downregulating the expression of *IL-6* due to the presence of PIGP polymer, and further anti-inflammatory evaluation will be studied in more detail in the next study.

### Osteogenic differentiation of C3H10T1/2 cells

**FIGURE 5:**
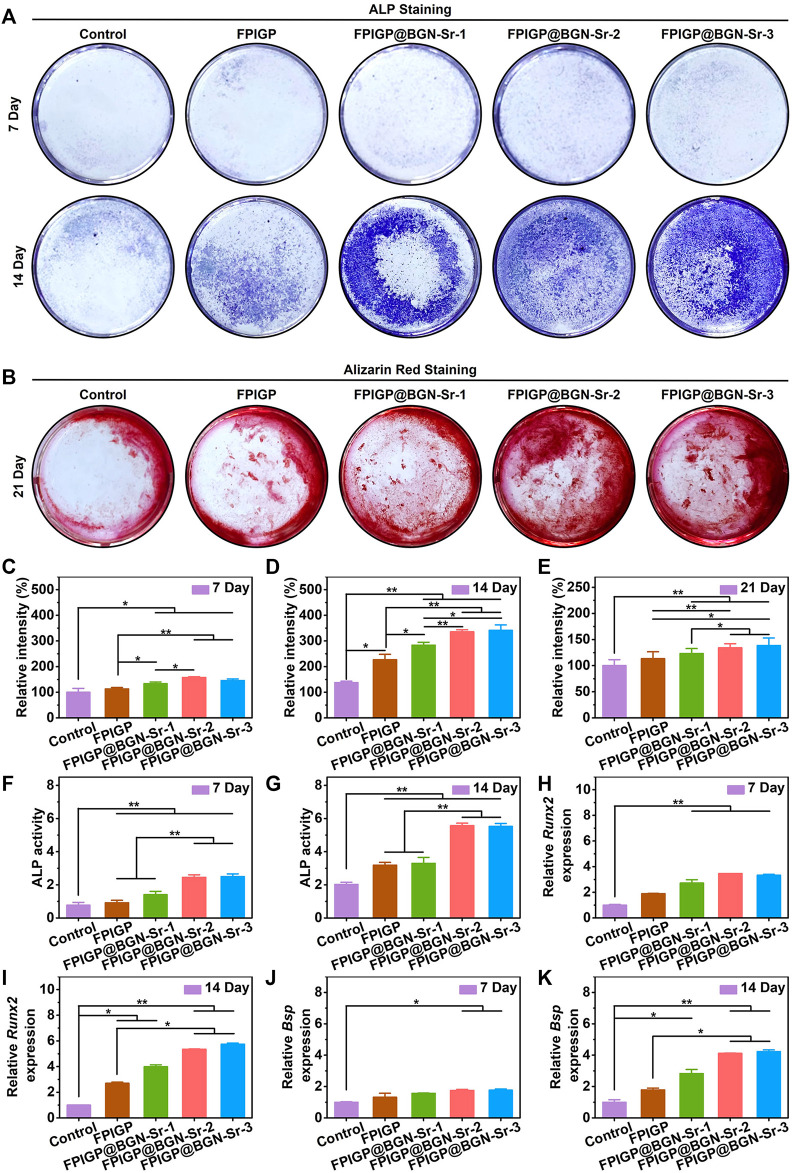
Osteogenic differentiation of C3H10T1/2 cells. **(A)** The ALP staining after incubation with hydrogels for 7 days and 14 days. **(B)** The alizarin red staining after incubation with hydrogels for 21 days. **(C**,**D)** The relative intensity based on ALP staining images of **(C)** 7 days and **(D)** 14 days. **(E)** The relative intensity based on alizarin red staining images of 21 days. **(F**,**G)** The ALP activity after incubation with hydrogels for **(F)** 7 days and **(G)** 14 days. **(H**,**I)** The relative *Runx2* expression after incubation with hydrogels for **(H)** 7 days and **(I)** 14 days. **(J**,**K)** The relative *Bsp* expression after incubation with hydrogels for **(J)** 7 days and **(K)** 14 days.

The osteogenic differentiation of C3H10T1/2 cells was evaluated by measuring ALP activity, biomineralization and osteogenic gene expression (Figure 5). ALP activity can be used as an early phenotypic marker of osteogenic differentiation, which can be evaluated through ALP staining and an ALP assay kit. For the ALP staining results, after C3H10T1/2 cells were cocultured with the hydrogels for 7 and 14 days, positive staining (blue) results were observed in each group, and the positive staining results were more pronounced at 14 days than at 7 days. Notably, more positive staining results were observed in the FPIGP@BGN-Sr hydrogels groups at 14 days than in the control group and FPIGP hydrogel group (Figure 5A). By analyzing the relative grayscale of the staining results, the FPIGP@BGN-Sr hydrogels groups showed higher relative intensity compared with the control group and FPIGP after cocultivation for 7 days (Figure 5C). After cocultivation for 14 days, the relative intensity of the FPIGP@BGN-Sr hydrogels groups were significantly higher than that of the control group and FPIGP hydrogel group, and there was a nonnegligible difference between the control group and FPIGP hydrogel group. Notably, compared to the FPIGP@BGN-Sr-1 hydrogel group, the FPIGP@BGN-Sr-2 and FPIGP@BGN-Sr-3 hydrogels groups exhibited higher relative intensity after 14 days of cocultivation. Meanwhile, no significant difference was observed between the FPIGP@BGN-Sr-2 and FPIGP@BGN-Sr-3 hydrogels groups (Figure 5D). In addition, the ALP activity was further measured to evaluate the osteogenic differentiation of C3H10T1/2 cells. The ALP activity of the FPIGP hydrogel group and FPIGP@BGN-Sr hydrogels groups were significantly higher than that of the control group after cocultivation for 7 and 14 days. It is worth noting that the ALP activity of the FPIGP@BGN-Sr-2 and FPIGP@BGN-Sr-3 hydrogels groups did not show a significant difference, but both were higher than that of the FPIGP and FPIGP@BGN-Sr-1 hydrogels groups, which was consistent with the ALP staining results (Figures 5F,G). The above results demonstrated that the FPIGP hydrogel had a weak promoting effect on ALP activity in the early stage of osteogenic differentiation of C3H10T1/2 cells, but the appropriate amounts of BGN-Sr nanoparticles significantly increased ALP activity.

Calcium nodule formation is a phenotypic marker of late osteogenic differentiation, which can be stained by alizarin red staining after cocultivation of C3H10T1/2 cells with the hydrogels for 21 days. Compared with the control group, the FPIGP hydrogel and FPIGP@BGN-Sr hydrogels groups showed more positive staining (red), and the FPIGP@BGN-Sr-2 and FPIGP@BGN-Sr-3 hydrogels groups had the most positive staining results (Figure 5B). By analyzing the relative grayscale of the staining results, the FPIGP@BGN-Sr hydrogels groups had higher relative intensity compared to the control and FPIGP hydrogels groups. No obvious difference in relative intensity was observed between FPIGP@BGN-Sr-2 and FPIGP@BGN-Sr-3 hydrogels groups, but their relative intensity was higher than that of the FPIGP@BGN-Sr-1 hydrogels group (Figure 5E). The above results indicated that FPIGP@BGN-Sr hydrogels could significantly promote calcium deposition and mineralization in the late osteogenic differentiation of C3H10T1/2 cells due to the suitable amounts of nanoparticles.

In addition, the promoting effect of FPIGP@BGN-Sr hydrogels on the osteogenic differentiation of C3H10T1/2 cells were further demonstrated by detecting osteogenic genes (*Runx2* and *Bsp*) expression. After cocultivation for 7 days, there was no significant difference in *Runx2* expression between the FPIGP hydrogel group and the control group. In contrast, *Runx2* expression in the FPIGP@BGN-Sr hydrogels groups was significantly increased compared with that in the control group. Moreover, there was no significant difference among the FPIGP@BGN-Sr hydrogels groups (Figure 5H). After cocultivation for 14 days, the *Runx2* expression in both the FPIGP hydrogel group and the FPIGP@BGN-Sr hydrogels groups were significantly higher than that in the control group. Notably, the *Runx2* expression in the FPIGP@BGN-Sr-2 and FPIGP@BGN-Sr-3 hydrogels groups were higher than that in the FPIGP hydrogel group, indicating that suitable amount of BGN-Sr nanoparticles can increase the *Runx2* expression (Figure 5I). For *Bsp* expression, compared with the control group after cocultivation for 7 days, only the FPIGP@BGN-Sr-2 and FPIGP@BGN-Sr-3 hydrogels groups had higher *Bsp* expression, while the FPIGP hydrogel group and FPIGP@BGN-Sr-1 hydrogel group showed no significant difference (Figure 5J). Compared with the control group after cocultivation for 14 days, the *Bsp* expression was significantly increased in the FPIGP@BGN-Sr hydrogels groups, but not in the FPIGP hydrogel group. It is worth noting that the *Bsp* expression in the FPIGP@BGN-Sr-2 and FPIGP@BGN-Sr-3 hydrogels groups were also higher than that in the FPIGP hydrogel group, indicating that the suitable addition of BGN-Sr nanoparticles can increase *Bsp* expression (Figure 5K). The results of the *Runx2* expression and the *Bsp* expression were consistent. The above results indicated that the FPIGP@BGN-Sr hydrogels could significantly promote the *Runx2* and *Bsp* expression during the osteogenic differentiation of C3H10T1/2 cells with the suitable amounts of BGN-Sr nanoparticles.

## Discussion

In this study, we developed an organic‒inorganic composite FPIGP@BGN-Sr hydrogel with excellent elasticity composed of DA-PF127 polymer, PIGP polymer and BGN-Sr nanoparticles to promote the osteogenic differentiation of C3H10T1/2 cells. The FPIGP@BGN-Sr hydrogels effectively maintained the activity and proliferation of C3H10T1/2 cells and promoted the osteogenic differentiation of C3H10T1/2 cells by enhancing ALP activity, promoting calcium deposition and extracellular matrix mineralization, and increasing the expression of osteogenic genes (*Runx2* and *Bsp*), which exhibited ideal application potential in bone tissue engineering.

Cell therapy is an important therapeutic strategy in bone tissue engineering. Exogenous cells, usually MSCs have been commonly used in bone tissue engineering (Moradi et al., 2018). C3H10T1/2 cells, as pluripotent progenitor cells, exhibit multiple differentiation pathways including osteogenic, chondrogenic, and myogenic differentiation under appropriate stimulation (Jasuja et al., 2005; Jiang et al., 2019; Zhang et al., 2020). For example, C3H10T1/2 cells can be induced to osteogenic differentiation by the stimulation of bone morphogenetic protein 9 (BMP-9), BMP-6, hydroxyapatite, and so on, which exhibit a wide range of applications in bone tissue engineering (Lin et al., 2009; Xu et al., 2012; Cai et al., 2020).

BGN nanoparticles play an important role in bone tissue engineering by enhancing the revascularization, cell adhesion and differentiation of MSCs and osteoprogenitor cells (Kaur et al., 2014). The release of silicate ions, calcium ions and phosphorus ions from BGN nanoparticles in the physiological environment can enhance the formation of hydroxyapatite and stimulate the expression of several genes including key osteoblast markers and extracellular matrix proteins (Dashnyam et al., 2017). Furthermore, our previous reports demonstrated that BGN nanoparticles can enhance the osteogenic differentiation of AMSCs by activating TGF-β/smad3 signaling pathway (Guo et al., 2018). In recent years, many trace elements have been introduced into BGN nanoparticles to improve their physical properties and achieve various functions including fluorescence, antibacterial, anti-inflammatory, promotion of bone remodeling and angiogenesis (Pantulap et al., 2021). Notably, Sr, as an osteophilic ion, can promote bone tissue regeneration by inducing bone matrix formation and inhibiting osteoclastogenesis (You et al., 2022). However, the further application of BGN nanoparticles requires delivery vehicles to achieve long-term retention and sustained release in bone tissue engineering *in vivo*. Among numerous delivery vehicles, biomedical hydrogels have been widely used due to their excellent 3D porous structure and extracellular matrix-like properties. The DA-PF127 polymer has photocuring ability by introducing propylene groups at both ends of the PF127 molecular chain (Gwon et al., 2018). As a metabolite of the body, itaconic acid has good anti-inflammatory ability, and its monoester also exhibits photocuring ability (Arnaud et al., 2020). β-GP, an osteogenic medium supplement and weak base, has been demonstrated to effectively increase the mechanical properties and osteogenic activity of polyesters due to its osteopromoting effect in its solute and conjugated forms (He et al., 2019).

Therefore, in our study, we utilized DA-PF127 polymer, PIGP polymer and BGN-Sr nanoparticles to prepare the FPIGP@BGN-Sr hydrogels with good elastic properties for promoting the osteogenic differentiation of C3H10T1/2 cells. Pure PF127 can form a thermosensitive hydrogel at a concentration of approximately 20% (wt/vol) without good elastic property (Wang C. G. et al., 2019). In contrast, the gel-forming concentration of the DA-PF127 hydrogel was only 10% (wt/vol), which greatly reduced the concentration of polymer. In addition, the DA-PF127 hydrogel exhibits good elastic property, and the propylene groups can undergo free radical polymerization with other reactive double bond groups. The PIGP polymer prepared by esterification has double bond groups and phosphate groups. On the one hand, the PIGP polymers can react with the DA-PF127 polymer to form the FPIGP hydrogel through free radical reactions. On the other hand, BGN-Sr nanoparticles can be incorporated into the FPIGP hydrogel network through coordination interactions with PIGP polymers. Notably, since BGN-Sr nanoparticles are involved in the network construction of the hydrogels, we prepared a series of hydrogels with different contents of BGN-Sr nanoparticles including FPIGP@BGN-Sr-1, FPIGP@BGN-Sr- 2 and FPIGP@BGN-Sr-3 to investigate their effect on the mechanical properties and biological activity of hydrogels. The results indicated that the maximum compressive strains of the FPIGP, FPIGP@BGN-Sr-1, FPIGP@BGN-Sr-2 and FPIGP@BGN-Sr-3 hydrogels were all close to 99%, and no obvious relaxation and hysteresis was observed at a compressive strain of 60% for 10 cycles, indicating that the doping amount of BGN-Sr nanoparticles had a negligible effect on the maximum compressive strain of the hydrogels. However, the doping amount of BGN-Sr nanoparticles had a significant effect on the compressive stress at a compressive strain of 60% and G′ of the hydrogels. In the biocompatibility evaluation, the FPIGP, FPIGP@BGN-Sr-1, FPIGP@BGN-Sr-2 and FPIGP@BGN-Sr-3 hydrogels all exhibited good hemocompatibility and cytocompatibility. Compared with the F@BGN-Sr hydrogel, the FPIGP@BGN-Sr hydrogel significantly downregulated *IL-6* expression in RAW 264.7 cells treated with LPS. For the osteogenic differentiation of C3H10T1/2 cells, the FPIGP hydrogel weakly promoted ALP activity and *Runx2* expression. Compared with the addition of BGN-Sr nanoparticles at a low concentration (25 μg/ml), the addition of higher concentrations (50 and 100 μg/ml) significantly enhanced the ALP activity, promoted calcium deposition and increased the *Runx2* and *Bsp* expression. It is worth noting that the promoting effect of FPIGP@BGN-Sr-2 and FPIGP@BGN-Sr-3 hydrogels on osteogenic differentiation was similar, indicating that BGN-Sr nanoparticles have a certain appropriate concentration for promoting osteogenic differentiation to further provide theoretical guidance for application in subsequent bone tissue engineering. According to the above results, the DA-PF127 and PIGP polymers mainly provide the host network for the composite hydrogel and endow the hydrogel with excellent elastic properties. PIGP polymers can confer certain anti-inflammatory ability to the FPIGP@BGN-Sr hydrogel by downregulating *IL-6* expression. BGN-Sr nanoparticles mainly provided the composite hydrogel with the ability to promote the osteogenic differentiation of C3H10T1/2 cells by enhancing ALP activity, promoting calcium deposition, and increasing the expression of *Runx2* and *Bsp*. However, the current experimental results only showed that the FPIGP@BGN-Sr hydrogel had excellent mechanical properties and could promote the osteogenic differentiation of C3H10T1/2 cells, but the specific mechanisms still need to be studied in the next study. In addition, the FPIGP@BGN-Sr hydrogel needs to be further functionalized to effectively embed cells and promote cell adhesion, proliferation and osteogenic differentiation, which laid the foundation for bone repair application *in vivo* to be a cell-loaded 3D printing bioink.

Compared with the currently reported hydrogels with promoting effect on the osteogenic differentiation of stem cells, the FPIGP@BGN-Sr hydrogel has several advantages. First, the preparation method of the FPIGP@BGN-Sr hydrogel was simple, and the monomers had good biocompatibility, which laid the foundation for large-scale production and clinical application. Second, the FPIGP@BGN-Sr hydrogel had good elastic property, which can not only avoid its fracture under load for *in vivo* bone repair but also expand its application in the biomedical field. Third, the FPIGP@BGN-Sr hydrogel had good hemocompatibility and cytocompatibility, which also beneficial for further clinical applications. Fourth, the FPIGP@BGN-Sr hydrogel can promote the osteogenic differentiation of C3H10T1/2 cells by enhancing ALP activity, promoting calcium deposition, and increasing the osteogenic genes expression, providing a promising biomaterial for subsequent *in vivo* bone tissue engineering. Fifth, the FPIGP hydrogel can be further functionalized due to the residual double bond and the BGN-Sr nanoparticles might be used to load small molecular active substances due to their porous structure, which exhibit desirable application potential in disease treatment and tissue engineering. Based on the above results, the FPIGP@BGN-Sr hydrogel was expected to be an excellent biomedical hydrogel for the treatment of diseases and regenerative medicine.

## Conclusion

In summary, in the present study we fabricated a new class of organic‒inorganic composite FPIGP@BGN-Sr hydrogels to promote the osteogenic differentiation of C3H10T1/2 cells. The FPIGP@BGN-Sr hydrogels exhibited excellent elastic properties including a maximum compressive strain close to 99% and fantastic resilience. The DA-PF127 polymer, PIGP polymer and BGN-Sr nanoparticles were synthesized by a simple, convenient and cost-effective method. Within the hydrogel formations investigated, the FPIGP@BGN-Sr-2 and FPIGP@BGN-Sr-3 hydrogels can significantly promote osteogenic differentiation of C3H10T1/2 cells by enhancing ALP activity, promoting calcium deposition and increasing osteogenic genes (*Runx2* and *Bsp*) expression. The development of FPIGP@BGN-Sr hydrogels strengthened the application of BGN inorganic nanoparticles in bone tissue engineering and expanded the repertoire of organic hydrogels with good elasticity. Therefore, the FPIGP@BGN-Sr hydrogels offer an option to meet the ever-increasing needs for functional biomaterials in tissue engineering and other biomedical applications.

## Data Availability

The authors acknowledge that the data presented in this study must be deposited and made publicly available in an acceptable repository, prior to publication. Frontiers cannot accept a manuscript that does not adhere to our open data policies.
