# Supplementary material for: Engineering elastic bioactive composite hydrogels for promoting osteogenic differentiation of embryonic mesenchymal stem cells
Source: Front Bioeng Biotechnol. 2022 Oct 12;10:1022153. doi: 10.3389/fbioe.2022.1022153 (PMC9596812; doi:10.3389/fbioe.2022.1022153)
Supplement: Supplementary file 2 [file DataSheet1.docx]

Supplementary Material

Engineering Elastic Bioactive Composite Hydrogels for Promoting Osteogenic Differentiation of Embryonic Mesenchymal Stem Cells

Min Wang^1, #^, Yi Guo^2, #^, Zexing Deng^3^, Peng Xu^1,*^

^1^Honghui Hospital, Xi'an Jiaotong University, Xi'an 710000, China

^2^Shaanxi Key Laboratory of Brain Disorders, Shaanxi Key Laboratory of Ischemic Cardiovascular Disease, Institute of Basic and Translational Medicine, Xi’an Medical University, Xi’an, 710021, China

^3^College of Materials Science and Engineering, Xi’an University of Science and Technology, Xi’an, 710054, China

^#^ Min Wang and Yi Guo contributed equally to this paper.

*** Correspondence:**Peng Xu
sousou369@163.com

Supplementary Figures


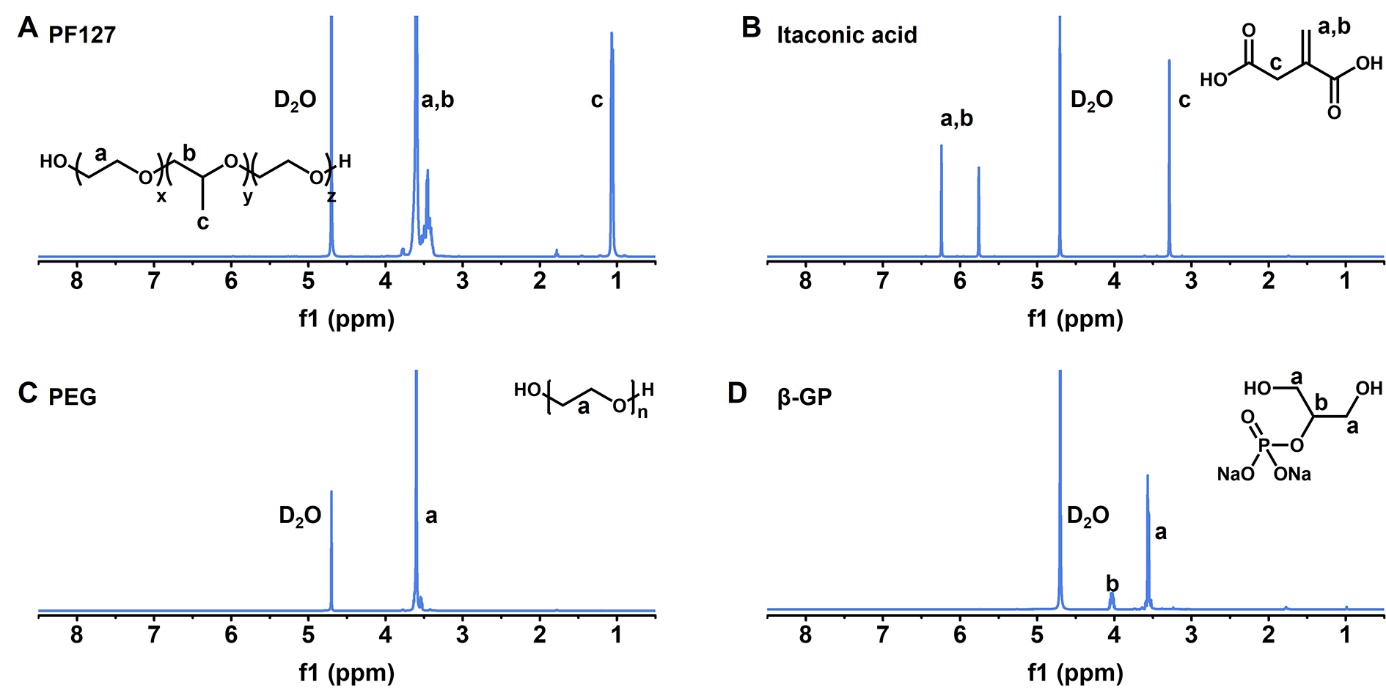


**Figure S1.** The ^1^H NMR spectra of **(A)** PF127, **(B)** itaconic acid, **(C)** PEG and **(D)** β-GP-Na.


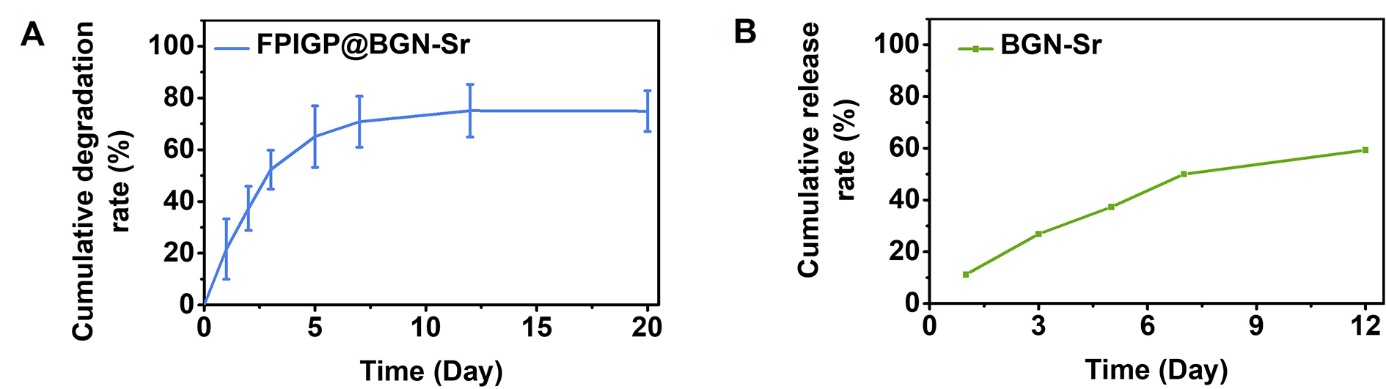


**Figure S2.** **(A)** The degradation of FPIGP@BGN-Sr hydrogel placed in PBS (pH 7.4) at 37 ℃; **(B)** The release of BGN-Sr from FPIGP@BGN-Sr hydrogel placed in PBS (pH 7.4) at 37 ℃.


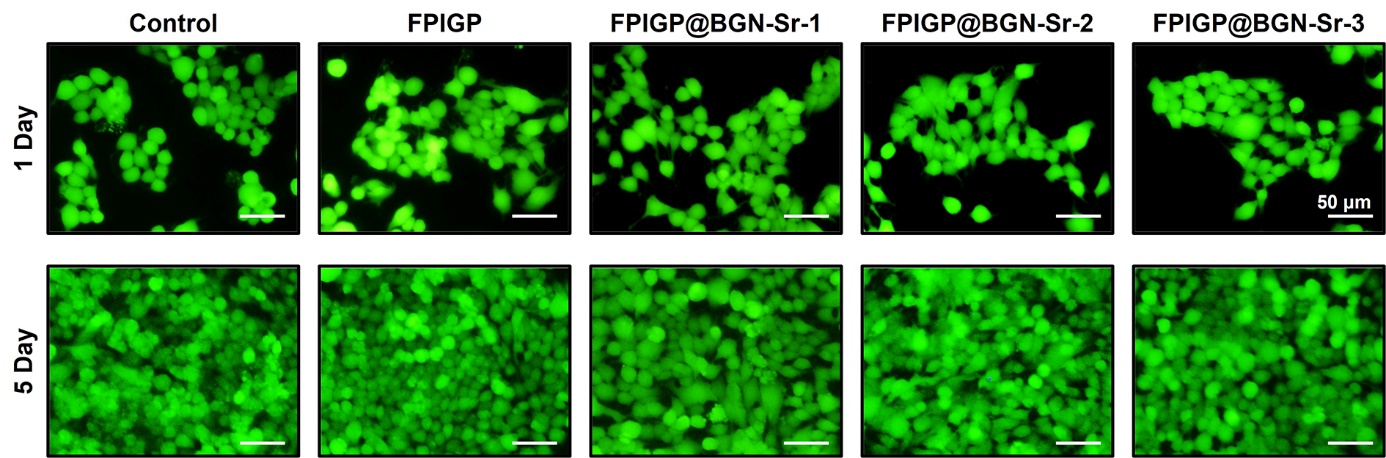


**Figure S3.** The live‒dead staining fluorescent images of C3H10T1/2 cells after incubation with hydrogels for 1 and 5 days.


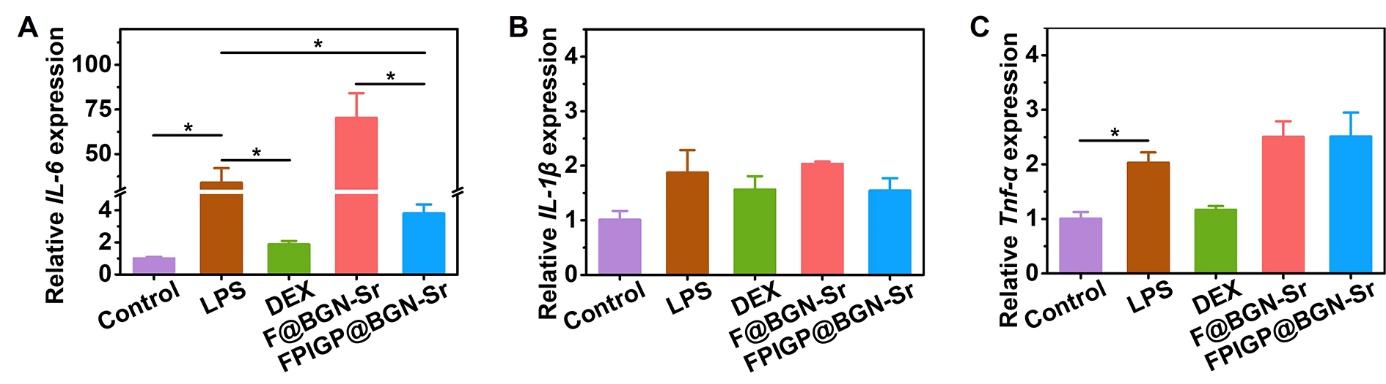


**Figure S4.** The relative (A) *IL-6*, (B) *IL-1β* and (C) *Tnf-α* expression in RAW 264.7 cells treated with LPS after incubation with hydrogels for 2 days.
